# Parotid area lymph node metastases from preliminarily diagnosed patients with nasopharyngeal carcinoma: report on tumor characteristics and oncologic outcomes

**DOI:** 10.18632/oncotarget.7677

**Published:** 2016-02-24

**Authors:** Yuanji Xu, Mingwei Zhang, Youping Xiao, Jingfeng Zong, Sufang Qiu, Penggang Bai, Yitao Dai, Lin Zhou, Xiaolin Chen, Wei Zheng, Yunbin Chen, Shaojun Lin, Jianji Pan

**Affiliations:** ^1^ The Shengli Clinical Medical College of Fujian Medical University, Fuzhou, China; ^2^ Department of Radiology, Teaching Hospital of Fujian Medical University, Fujian Provincial Cancer Hospital, Fuzhou, China; ^3^ Department of Radiation Oncology, Teaching Hospital of Fujian Medical University, Fujian Provincial Cancer Hospital, Fuzhou, China; ^4^ Fujian Provincial Key Laboratory of Translational Cancer Medicine, Fuzhou, China

**Keywords:** nasopharyngeal carcinoma, parotid area lymph node metastases, tumor characteristics, oncologic outcomes

## Abstract

The parotid area lymph node (PLN) is an uncommon site of metastasis originating from nasopharyngeal carcinoma (NPC). The study aimed to investigate clinical characteristics and outcomes of patients with preliminarily diagnosed NPC with PLN metastases. Here we retrospectively reviewed Magnetic resonance imaging (MRI) scans of 2221 patients with untreated nonmetastatic NPC who received intensity-modulated radiation therapy (IMRT). Finally, 64 (2.9%) patients were identified with PLN metastases, of which, 34 received PLN-sparing IMRT and 30 received PLN-radical IMRT. We also found that 42.2% had N3 disease and 95.3% had stages III-IVb. PLN metastases on MRI were characterized by ipsilateral retropharyngeal lymph node (RLN) or level II nodal extracapsular spread (ECS), ipsilateral giant cervical nodes, ipsilateral parapharyngeal extension, or solitary parotid metastasis. The 5-year overall survival, distant metastasis-free survival, regional relapse-free survival, and parotid relapse-free survival rates were 70.4%, 64.3%, 76.7%, and 87.9%, respectively. Distant metastases were the main cause of treatment failure and death. Using PLN-sparing IMRT, sparing PLN with minimal axial diameter of <10 mm, could increase the risk of parotid recurrence. However, it was not an independent prognostic factor. N classification and concurrent-based chemotherapy were almost statistically significant for distant failure and death. Overall, we demonstrated that the PLN metastases might be derived from RLN or level II nodal ECS, giant cervical nodes in a retrograde fashion, or parapharyngeal extension. Sparing PLN of <10 mm by IMRT should consider the risk of parotid recurrence. Distant metastases remained the dominant treatment failure. Further effective systemic chemotherapy should be explored.

## INTRODUCTION

Nasopharyngeal carcinoma (NPC) is the most commonly diagnosed malignant cancer in Southern China and parts of Southeast Asia, and radiotherapy has been the mainstay treatment for NPC [1]. NPC is characterized by high incidence of enlarged cervical nodes at presentation, but it is unusually involved by parotid area lymph node (PLN) metastases with the incidence rate of only 0.6%-3.4% [2-5]. Compared with conventional radiotherapy, intensity-modulated radiation therapy (IMRT) not only provided improved locoregional control but also had better protection for parotid glands to reduce xerostomia [6-7]. However, with the prevailing use of parotid-gland-sparing IMRT, one concern is that the reduction of dose to parotids might lead to a deficient radiation exposure to lymph nodes in and around the parotid gland.

In 2008, Cannon and Lee first reported on two patients with NPC who developed periparotid failure after definitive parotid-gland-sparing IMRT [8]. Subsequently, three patients with NPC were found to have periparotid recurrence after parotid-gland-sparing radiotherapy by Lin et al, who considered that advanced tumor stage, subclinical metastasis at the parotid area, and over-protection of the parotid gland may be correlated with the development of recurrence [9]. In 2013, ten patients with NPC developed periparotid recurrence after parotid-gland-sparing IMRT by Cao et al, suggesting that the over-protection of ipsilateral parotid of the primary tumor center especially for NPC with lateral retropharyngeal lymph nodes (RLNs) could have contributed to this recurrence [10].

However, these retrospective studies were confined to a small cohort of periparotid recurrent patients to explore the clinical characteristics of potential PLN metastases in preliminarily diagnosed patients with NPC, with little attention paid to patients with NPC with definite metastases to the parotid region at initial diagnosis [2-3]. In addition, a discrepancy existed among clinicians about sparing or irradiating PLN with the minimal axial diameter (MID) of <10mm in imaging findings of patients treated with IMRT, because periparotid recurrence is an exceedingly uncommon pattern of failure after parotid-gland-sparing radiation for NPC [8-11], and whether over-protection of parotid glands would influence the prognosis remains largely unknown [3].

The aim of the present study is to investigate the tumor characteristics of patients with preliminarily diagnosed NPC with PLN metastases using magnetic resonance imaging (MRI) to enhance the awareness of the potential of NPC to metastasize to the parotid region and to report their oncologic outcomes to provide a reference on treatment.

## RESULTS

### Treatment outcome and the characteristics of parotid area lymph node spread

For the entire cohort, the 5-year overall survival (OS), distant metastasis-free survival (DMFS), regional relapse-free survival (RRFS), and parotid relapse-free survival (PRFS) rates were 70.4%, 64.3%, 76.7%, and 87.9%, respectively. A total of 19 patients died (13 of distant metastasis, 1 of local recurrence, 2 of treatment complications, and 3 of unknown reasons), 20 developed distant metastases, and 2 developed local recurrences. A total of 10 patients developed regional recurrence, of which 6 patients were found to develop periparotid recurrence and the most common sites involved were: subcutaneous pre-auricular site (1 case), the superficial intraparotid site (4 cases), and both the superficial and deep intraparotid sites(1 case). The median periparotid nodal recurrence period was 21 months (range, 6-49 months). After periparotid recurrence, all six patients were treated with curative treatment to recurrent PLN including radical surgery with or without chemotherapy, or definitive radiotherapy with or without chemotherapy.

**Figure 1:**
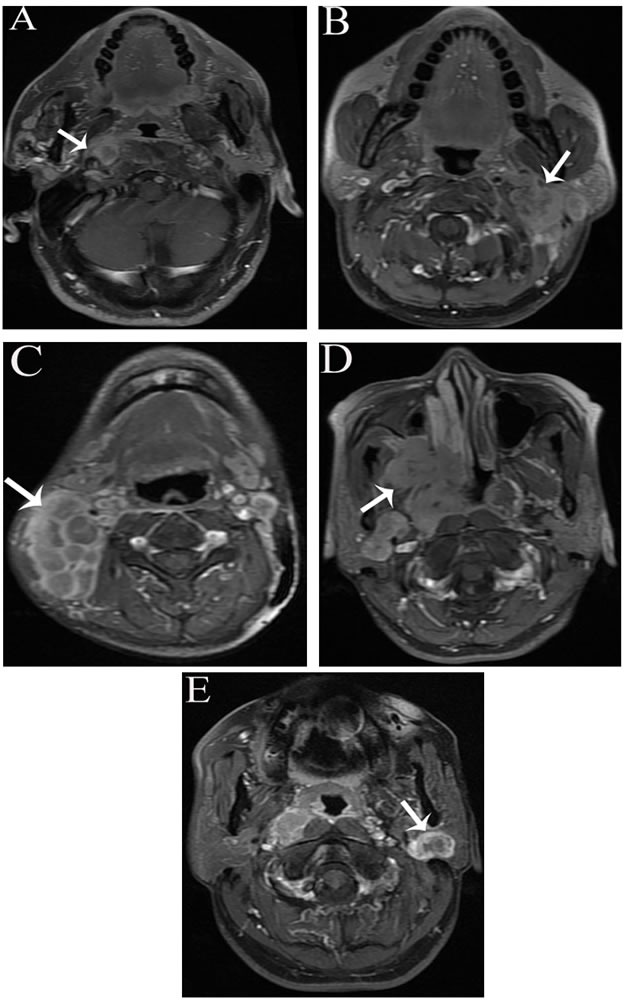
The characteristics of PLN spread on axial contrast-enhanced T1 fast spin-echo fat-suppressed (FSE fs) MRI **A.** Ipsilateral retropharyngeal lymph nodal extracapsular spread; **B.** ipsilateral level II nodal extracapsular spread; **C.** ipsilateral giant cervical nodes; **D.** ipsilateral extensive parapharyngeal space involvement; and **E.** solitary parotid lymphadenopathy.

**Figure 2:**
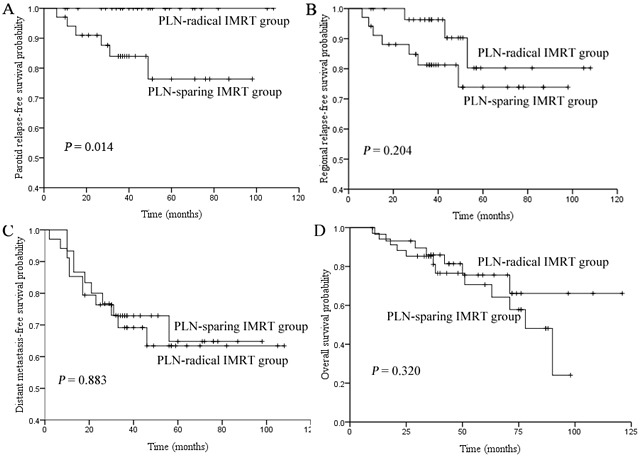
Comparison of the 5-year parotid relapse-free survival (PRFS) **A.** regional relapse-free survival (RRFS) **B.** distant metastasis-free survival (DMFS) **C.** and overall survival (OS) **D.** rates between PLN-sparing IMRT group and PLN-radical IMRT groups.

**Figure 3:**
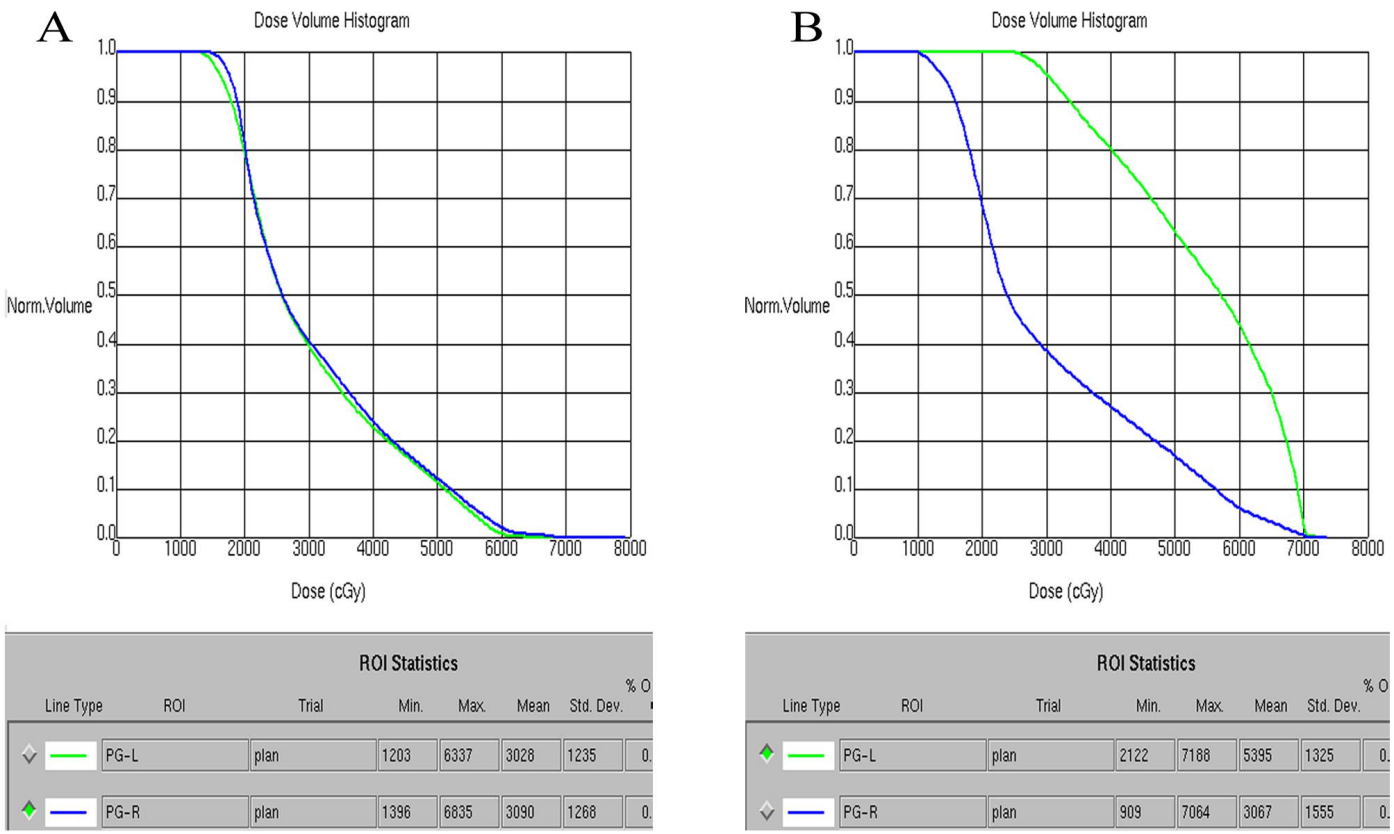
The dose volume histogram (DVH) chart of parotid glands in two patients with parotid area lymph nodes (PLNs) metastasis treated with intensity-modulated radiotherapy (IMRT) **A**. One patient with right PLN metastasis treated with PLN-sparing IMRT. **B**. The other patient with left PLN metastasis treated with PLN-radical IMRT.

Of the 64 patients, 27 (42.2%) had N3 disease and 61 (95.3%) had stages III-IVb. According to the imaging features on MRI, the characteristics of PLN spread could be categorized into four groups: (1) ipsilateral adjacent lymphatic drainage lymphadenopathy (RLN or level II) accompanied by extracapsular spread (ECS) (Figures 1A and 2B); (2) ipsilateral giant cervical lymphadenopathy (Figure 1C); (3) ipsilateral extensive parapharyngeal space involvement (Figure 1D); and (4) solitary parotid lymphadenopathy (Figure 1E). Information on other variables including nodal size, laterality, location, number and primary tumor center are also shown in Table 1.

**Table 1:** Characteristics of parotid area nodal spread in 64 preliminarily diagnosed NPC patients

Characteristics	No. of Patients (%)
Type	
Ipsilateral RLN or Level II lymphadenopathy with ECS	47 (73.4%)
Ipsilateral giant cervical lymphadenopathy	11 (17.2%)
Ipsilateral extensive parapharyngeal space involvement	10 (15.6%)
Solitary parotid lymphadenopathy	6 (9.4%)
Size	
MID <10mm	39 (60.9%)
MID≥10mm	25 (39.1%)
Laterality	
Unilateral PLN	62 (96.9%)
Bilateral PLN	2 (3.1%)
Location	
subcutaneous pre-auricular site	3 (4.7%)
superficial intraparotid	37 (57.8%)
deep intraparotid	4 (6.3%)
Subparotid	5 (7.8%)
Multiple sites	15 (23.4%)
Number	
Single	39 (60.9%)
Multiple	25 (39.1%)
Primary tumor center	
Ipsilateral	40 (62.5%)
Middle	12 (18.8%)
Contralateral	12 (18.8%)

Abbreviations: RLN, retropharyngeal lymph node; ECS, extracapsular spread; MID, minimal axial diameter; PLN, parotid area lymph node.

### Comparison of treatment outcomes between the PLN-sparing and PLN-radical IMRT groups

To compare the treatment outcomes of patients with initial PLN metastases between the PLN-sparing and PLN-radical IMRT groups, pretreatment and treatment characteristics needed to be firstly evaluated (as shown in Table 2). The characteristics of the two groups were comparable except the PLN size. The PLN-sparing IMRT group was characterized by PLN with MID of <10 mm.

**Table 2:** Comparison of clinical characteristics in 64 preliminarily diagnosed NPC patients between PLN-sparing and PLN-radical IMRT group

	PLN-sparing IMRT group	PLN-radical IMRT group	*P* value
Cases (n)	34	30	
Age (y)			0.268
≤ 50	22	20	
> 50	12	10	
Gender			0.285
Male	31	24	
Female	3	6	
Parotid nodal size			<0.001
MID < 10mm	34	5	
MID ≥ 10mm	0	25	
T stage			0.258
T1	7	2	
T2	4	8	
T3	14	11	
T4	9	9	
N stage			0.217
N1	6	10	
N2	14	7	
N3 (N3a +N3b)	14	13	
Clinical stage			0.841
II	1	2	
III	14	12	
IV (IVa +IVb)	19	16	
Chemotherapy regime			0.316
Concurrent-based	14	17	
Non-concurrent-based	20	13	

Abbreviations: PLN, parotid area lymph note; minimal axial diameter.

All six patients with periparotid recurrence were in the PLN-sparing IMRT group, which had a significantly lower 5-year PRFS compared with that in the PLN-radical IMRT group (76.3% vs. 100%, *P* = 0.014), as shown in Figure 2A. However, no statistically significant difference was reported in the 5-year RRFS (73.9% vs. 80.2%, *P* = 0.204), DMFS (64.8% vs. 63.4%, *P* = 0.833), and OS (64.8% vs. 71.1%, *P* = 0.320) between the two groups, as shown in Figure 2B, 2C, and 2D, respectively.

### Independent prognostic factors for patients with preliminarily diagnosed NPC with PLN metastases

To explore the significance of potential prognostic factors, the following covariables were involved in the Cox proportional hazards model: sex (male vs. female), age (≤50 years vs. >50 years), T classification, N classification, clinical stage, radiotherapy (PLN-sparing IMRT vs. PLN-radical IMRT), chemotherapy regime (concurrent-based vs. nonconcurrent-based), and chemotherapy cycles (≤3 cycles vs. >3 cycles). As shown in Table 3, using PLN-sparing IMRT failed to show a significant positive trend for all endpoints including periparotid failure. However, N classification was statistically significant for both distant failure (*P* = 0.013) and death (*P* = 0.035), and concurrent-based chemotherapy was an adverse prognostic factor for OS (*P* = 0.026) and a borderline significance for DMFS (*P* = 0.081).

**Table 3:** Multivariate analysis of potential prognostic factors in patients with parotid area nodal spread

Endpoint	Factor	*P* value	HR	95%CI for HR
Distant failure	N classification	0.0130	2.2812	1.190-4.373
	Chemotherapy regime	.0810	2.274	0.904-5.720
Death	Age	0.001	5.433	1.971-14.979
	N classification	0.035	1.928	1.046-3.553
	Chemotherapy regime	0.026	3.022	1.143-7.993

Abbreviations: HR, Hazard ratio; CI, Confidence interval.

## DISCUSSION

When treating patients with NPC, IMRT undoubtedly provides immense protection for salivary function by sparing parotid glands [6-7]. In addition, parotid-gland sparing IMRT could only result in sparse periparotid recurrence [8-11]. Hence, some patients with PLN of <10mm on MRI were inclined to use PLN-sparing IMRT instead of PLN-radical IMRT in the case of severe salivary damage in clinical practice. Currently, a paucity of knowledge exists on the clinical characteristics and outcomes of patients with NPC with initial PLN metastases [1, 3]. The present study represents a novel research to analyze tumor characteristics, compare treatment outcomes between the PLN-sparing IMRT and PLN-radical IMRT groups, and explore potential prognostic factors of patients with NPC with initial PLN metastases based on MRI in the literature. The study found that the initial PLN metastasis was an uncommon pattern of regional metastasis for NPC with the incidence of only 2.9%. PLN metastases could be characterized by ipsilateral RLN or level II nodal ECS, giant cervical nodes, extensive parapharyngeal space involvement, or solitary parotid metastasis. PLN-sparing IMRT could increase the risk of periparotid recurrence compared with PLN-radical IMRT. However, it was not an adverse prognostic factor for all survival endpoints; N classification remained statistically significant for both DMFS and OS, and concurrent-based chemotherapy was an independent prognostic factor for OS and of borderline significance for DMFS.

The parotid group nodes receive lymphatic drainage mostly from cutaneous sites such as the frontal and temporal skin, eyelids, external acoustic meatus, and root of the nose, but other regions, including the nasopharynx, are also potential foci [19, 21]. The initial PLN metastasis is rare in NPC, and its diagnostic rates by MRI ranged from 1.2% to 2% [2-3]. Similarly, the current study was also mainly based on MRI, and the incidence of PLN metastasis was 2.9% (64/2221), which was slightly higher than the rates reported in the former two studies. It should be noted that initial PLN metastasis in NPC indeed presented as an unusual pattern of regional metastasis. However, no unified imaging diagnostic criteria have been reported for PLN metastasis with NPC at present [3]. Any parotid lymphadenopathy in spite of nodal size was even considered to be metastatic PLN in some treatment centers. In fact, unavoidable false-negative and false-positive diagnoses might be obtained as a result of seldom acquisition of PLN pathological confirmation in clinical practice. Therefore, this retrospective study was performed not only to take cervical lymph nodes (CLN) criteria as the reference standard for PLN metastasis but also combined with MRI follow-up to ensure PLN with partial response (PR) or completed response (CR) 3 months after completing radiotherapy, which would no doubt improve the diagnostic accuracy.

According to the four groups of tumor characteristics of initial PLN metastases with NPC on MRI in this study, three possible mechanisms exist to explain the metastasis of NPC to PLN. First, tumor can spread to the parotid region via aggressive ECS from ipsilateral RLN or level II lymphadenopathy. NPC had a high frequency of enlarged cervical node metastasis, and RLN and level II nodes were commonly thought to be the first nodal stations for regional spread with an overall probability of 69.4% and 70.4%, respectively [22]. These two-level nodes were in proximity to the parotid gland, and tumor can easily extend into the parotid gland as a result of extracapsular rupture. In this series, most of the patients (73.4%) with ipsilateral RLN or level II nodes presented with ECS on MRI, which may predominantly contribute to the development of PLN metastasis. Second, tumor may reach the parotid gland from ipsilateral giant lymphadenopathy in a retrograde fashion. The normal lymphatic drainage of the neck could be disrupted by giant cervical nodes, leading to uncommon retrograde metastases to PLN. Finally, 10 patients (15.6%) had ipsilateral extensive parapharyngeal space involvement. When parapharyngeal space was extensively involved, tumor could spread posterolaterally across the space between the pterygoid process and the medial pterygoid muscle to the parotid gland, directly resulting in intraparotid lymph node metastasis. It is hard to explain why a small number of patients (9.4%) only presented with solitary parotid lymphadenopathy. Anyhow, the clinicians should enhance the awareness of potential metastasis to parotid group nodes through these three possible mechanisms, which would provide reference information for the future definition of the parotid group as clinical target volume (CTV) based on the 2013 consensus guidelines for the delineation of neck CTV [19].

In this series, the patients with initial PLN metastases were treated with either PLN-sparing IMRT or PLN-radical IMRT. The PLN-spring IMRT group was shown to have a significant higher rate of periparotid recurrence compared with the PLN-radical IMRT group (*P* = 0.014), and all six patients with periparotid recurrence were found in the PLN-sparing IMRT group. The study demonstrated that using PLN-radical IMRT could reduce the risk of parotid recurrence. The characteristics of the 2 groups were comparable except the parotid nodal size, and all 34 patients of the PLN-sparing group were characterized by PLN with MID of <10mm (Table 2). Therefore, such patients were candidates to receive adequate attention regardless of the low incidence of ECS or central necrosis in the lymph node with an MID of < 10mm on MRI [23-24]. It is necessary to perform fine-needle aspiration cytology (FNAC) and/or position emission tomography / computed tomography (PET/CT) in patients with suspicious PLN metastases to make a definite diagnosis in the case of PLN-sparing IMRT. However, there were no statistically significant difference was observed in the 5-year RRFS, DMFS, and OS between the two groups, because the patients with periparotid recurrence in this study could be treated with successful salvage strategies, which may make little impact on the prognosis.

For have further multivariate analyses, PLN-sparing IMRT was no longer an adverse prognostic factor for periparotid failure. However, the N classification was of statistical significance for both distant failure (*P* = 0.013) and death (*P* = 0.035), and concurrent-based chemotherapy was found to be an independent prognostic factor for OS (*P* = 0.026) and of borderline significance for DMFS (*P* = 0.081). In addition, 2 local recurrences, 10 regional recurrences, and 20 distant metastases were reported in this series. Distant metastases were the dominant treatment failure and death. These results hinted that concurrent platinum-based chemotherapy was of great importance to reduce distant failure and prolong survival, and effective systemic therapy for advanced N disease was in demand. In the IMRT era, the addition of concurrent chemotherapy (CCRT) has proved its superiority over radiotherapy alone to reduce treatment failure and improve survival, and CCRT is still considered a standard modality for locoregionally advanced NPC [25-27]. Considering that 95.4% of patients were classified as locoregionally advanced stages (III-IVb) in this study, concurrent-based chemotherapy would no wonder play a vital role in reducing the distant failure and death. However, distant metastasis remained the most difficult treatment challenge for locoregionally advanced patients with IMRT plus concurrent chemotherapy. In a prospective, multicentric clinical study conducted by Wu et al, distant metastasis was the main cause of treatment failure and death, and N classification was an independent prognostic factor for RRFS, DMFS, and OS in locoregionally advanced NPC treated with IMRT and concurrent cisplatin chemotherapy [28]. Therefore, a pressing need exists to explore more effective systemic chemotherapy. Hui et al in a randomized phase II trial showed that concurrent cisplatin radiotherapy with neoadjuvant docetaxel and cisplatin provided better progressive-free survival (PFS) and OS as compared with concurrent cisplatin radiotherapy alone in locoregionally advanced NPC [29]. A phase III study to verify this neoadjuvant-concurrent strategy is warranted. Considering that more than 43.4% of patients had N3 disease in this series, the addition of adjuvant chemotherapy to neoadjuvant-concurrent strategy might have survival benefit. Xu et al in their retrospective study demonstrated that 37 patients with NPC with N3 disease treated with CCRT followed by adjuvant chemotherapy acquired better 5-year DMFS and OS rates compared with 15 such patients with NPC with N3 disease treated with CCRT alone. Large multicentric randomized clinical trials were needed for further verification [30].

One limitation of this present study was the lack of nodal biopsy confirmation except in only one case. Patients suspected of PLN metastasis could be proved by pathology to guide further treatment. Additionally, because some patients were involved into the randomized clinical trials to have certain chemotherapy regimens, or some chemotherapy regimens were conducted at the discretion of attending physicians because of individual cases, only 31 patients (48.4%) received concurrent platinum-based chemotherapy considering that most patients (95.3%) were diagnosed as locoregionally advanced stage. However, CCRT is still regarded as the current standard treatment for locoregionally advanced NPC in the IMRT era. It is necessary to analyze the prognosis of the initial PNL metastases of NPC with the increasing number of CCRT for advanced disease. Finally, the current study represents a retrospective study and the number of patients with NPC with initial PLN metastases was relatively small; hence, a large, prospective, and randomized clinical trial is warranted.

At present, due to a low incidence of initial PLN metastases in NPC, the prognostic significance of PLN metastases in preliminarily diagnosed patients with NPC remains largely unknown, and the parotid node group is not included in the current edition of the International Union Against Cancer/ American Joint Committee on Cancer (UICC/AJCC) staging system [3, 12-13]. The present study showed that only 2.9% developed PLN metastases at initial diagnosis, but their prognosis was poor, with the 5-year OS, DMFS, RRFS of only 70.4%, 64.3%, and 76.7%, respectively. In clinical practice, PLN metastasis is often classified as CLN metastasis, thus it may be appropriate to be included in the N stage. A previous study showed that the 5-year DMFS for N3a and N3b treated with IMRT based on MRI were 70.2% and 61.7%, respectively [15], which were similar with the 5-year DMFS for PLN metastases in the present study. Furthermore, 42.2% of patients with PLN metastases in the present study had N3 disease, and the 5-year OS for patients with PLN metastases in our present study was similar to the 5-year DSS for patients with stage IVb in our previous study (70.4% vs. 68.9%) [15]. Therefore,it may be reasonable to classify PLN metastases as N3 disease in the current staging system. Recently, Zhang et al firstly reported that PLN metastasis was significantly associated with poor DMFS, and it had a similar higher hazard ratio (HR) to N3 disease [3]. However, only 10 patients with PLN metastases receiving PLN-radical IMRT were included in the prognostic analyses, which might not have enough persuasive power. In addition, other patients with PLN metastases receiving PLN-sparing IMRT should also be included in prognostic analyses considering that using PLN-sparing IMRT was not an independent prognostic factors for all survival endpoints in the current study. Hence, the next strategy is to evaluate the prognostic significance of all PLN metastases compared with different N classification of a large cohort of patients in an attempt to establish its status in the current staging system.

In conclusion, the PLN metastases in NPC might be derived from RLN or level II nodal ECS, giant cervical nodes in a retrograde fashion, and infiltration of parapharyngeal extension. Sparing PLN of <10 mm on MRI by IMRT could increase the risk of parotid recurrence. Distant metastases remained the dominant treatment failure for initial PLN metastases with NPC. Further effective systemic chemotherapy should be explored.

## MATERIALS AND METHODS

### Patients and pretreatment evaluation

This retrospective study was performed with the approval of the Institutional Review Board of Fujian Provincial Cancer Hospital. The charts of 2221 patients with pathologically proven and newly diagnosed nonmetastatic NPC who underwent IMRT in Fujian Provincial Cancer Hospital between June 2005 and December 2012 were reviewed. All patients underwent scanning of the nasopharynx and neck by MRI. Among them, 64 (2.9%) patients were found to develop PLN metastases by MRI. Of the 64 patients, 55 were males and 9 were females the median age was 46 years (range, 16 to 79 years). Histologically, 96.9% of the patients had WHO type III disease and 3.1% had WHO type II disease. The pretreatment evaluation of all patients was accomplished according to the institutional protocol [12], and the disease staging was performed according to the seventh edition of the International Union Against Cancer/American Joint Committee on Cancer (UICC/AJCC) staging system for NPC [13-14]. None of these patients had previously experienced surgical treatment or radiotherapy to the head or neck.

### Imaging protocol, assessment, and diagnostic criteria

The MRI scanning protocol has been published in previous studies by the authors of this study [15-16]. Briefly, all scanning sequences extended from the central temporal lobe to the thoracic outlet. Between June 2005 and December 2010, all patients underwent MRI with a 1.5-T system (Singa EXCITE III HD, American GE Company, WI, USA) and an eight-channel neurovascular transceiver coil. Seven scanning sequences including axial and sagittal T1 fast spin-echo (FSE), axial proton density fat-suppressed (PD fs), coronal T1 short-tall inversion recovery (STIR), axial and coronal T1 enhanced FSE fs, and diffusion weighted imaging (DWI) were generally obtained in MRI scans. From January 2011 to December 2012, all patients underwent MRI on a 3.0-T whole-body multi-transmit scanner (Achieva TX, Philips Healthcare, Best, The Netherlands) and a 16-channel neurovascular transceiver coil. Routine MRI scans contained axial and sagittal T1-weighted image (T1WI), axial and oblique coronal T2WI (with SPIR technique), axial and oblique coronal T1WI (with SPIR technique) after contrast injection, and DWI. All MRI scans were independently reviewed by two radiologists who specialized in head and neck cancers, and the differences were resolved by consensus. Other imaging tests or studies such as PET were used at the attending physician's discretion.

MRI diagnostic criteria for lymph node metastasis were indicated by the literature. Criteria for RLN involvement included lateral RLN with an MID of ≥5 mm, whereas any node in the median retropharyngeal group was considered metastatic [17]. CLNs were regarded as metastases in the presence of central necrosis or contrast-enhanced rim or extracapsular spread (ECS) irrespective of size, or if their MID was of ≥10 mm, or if there was a group of three or more nodes of borderline size with MID of 8-10 mm [5, 18]. According to the 2013 Consensus Guidelines for nodal levels, the parotid node group was defined as level VIII, which included the subcutaneous preauricular nodes, the superficial and deep intraparotid nodes and the subparotid nodes [19]. At present, no unified MRI diagnostic criteria has been reported for PLN metastasis; hence CLN criteria was taken as the reference standard and some supplements were made for parotid node group metastasis, which should fulfill the following criteria : (1) a parotid lesion reached CLN metastatic criteria on MRI, and it achieved CR or PR based on Response Evaluation Criteria in Solid Tumors 1.1 criteria [20] 3 months after completing radiotherapy on routine MRI follow-up; (2) or an unsuspicious pre-existing parotid lesion developed recurrence during the follow-up period. (3) or a parotid lesion was confirmed with pathology by FNAC.

### Treatment

All patients underwent definitive IMRT. Details about the IMRT used at Fujian Provincial Cancer Hospital have been previously described [12]. Of the 64 patients, PLNs of 34 patients (53.1%) were received by sparing a prescribed dose that was comparable with the dosage delivered to parotid glands (PLN-sparing IMRT group), and the volume of each parotid receiving a dose greater than 26-30Gy was limited to less than 50% (as shown in Figure 3A). However, 30 patients (46.9%) with PLNs were received by a radical prescribed dose that was identical to the dose delivered to CLNs with 66-68.20Gy in 30-31 fractions to the PTV of GTV-N (PLN-radical IMRT group), and the volume of ipsilateral parotid receiving a dose greater than 26-30Gy was much more than 50%, leading to the complete sacrifice of the ipsilateral parotid (as shown in Figure 3B).

All 64 patients were staged as II-IVb, and 61 (95.3%) received platinum-based chemotherapy: the sequence given was induction in 6 (9.4%), concurrent in 2 (3.1%), adjuvant in 1 (1.6%), induction-concurrent in 12 (18.8%), induction-adjuvant in 22 (33.4%), and induction-concurrent-adjuvant in 18 (28.1%). When possible, salvage treatments such as afterloading, surgery, and chemotherapy were given to patients who developed recurrence or when persistent disease was documented.

### Follow-up and statistical analysis

The median follow-up time for the whole group was 44 months (range, 10-121 months). The pretreatment and treatment characteristics between PLN-sparing and PLN-radical IMRT groups were compared using the chi-square test (or Fisher's exact test if the expected number was less than five in any cell). The duration of OS, DMFS, RRFS, and PRFS was calculated from the start of diagnosis to death, distant failure, regional failure, and parotid recurrence, respectively. If patients were still alive at the end of the follow-up, the survival duration was censored. The survival rates were estimated using the Kaplan-Meier method and compared using the log-rank test. Multivariate analyses with the Cox proportional hazards model were used to determine the significance of the independent prognostic factors for patients with NPC with initial PLN metastases. A two-tailed *P* value of <0.05 was considered to be statistically significant. All analyses were performed using the SPSS version 17.0 software (SPSS, Inc., Chicago, IL, USA).
